# Diagnostic Performance of Ultrasound to Evaluate Mild Hip Osteoarthritis: Comparison With Radiography and MRI

**DOI:** 10.1002/jcu.70086

**Published:** 2025-09-20

**Authors:** Antti Kemppainen, Saana Kaartinen, Mika T. Nevalainen

**Affiliations:** ^1^ Research Unit of Health Sciences and Technology, Faculty of Medicine University of Oulu Oulu Finland; ^2^ Department of Diagnostic Radiology Oulu University Hospital Oulu Finland; ^3^ Medical Research Center Oulu University of Oulu and Oulu University Hospital Oulu Finland

**Keywords:** articular, cartilage, diagnostic imaging, hip, hip joint, musculoskeletal diseases, osteoarthritis, ultrasonography

## Abstract

**Purpose:**

To study the diagnostic performance of ultrasound (US) in mild hip osteoarthritis (HOA) compared to conventional radiography (CR) and magnetic resonance imaging (MRI).

**Methods:**

Fifty‐eight patients referred to CR for suspected HOA with at least unilateral Kellgren‐Lawrence (KL) 2 change in the CRs were recruited. Bilateral hip US and MRI (*n* = 116) were performed, and patients filled the Visual Analog Scale (VAS) bilaterally. A sum US score was formed, and its correlation to KL grades and VAS scores was tested. Descriptive statistics, Chi square and McNemar's test, Spearman correlation, and linear regression analysis were applied as statistical techniques.

**Results:**

US and CR showed similar moderate diagnostic performance compared to MRI with moderate correlation (*r* = 0.449) between US sum score and KL grades. With pain associations, US sum score showed an OR = 1.725 (CI 1.169–2.546) and KL grades an OR = 2.058 (CI 1.038–4.082).

**Conclusion:**

US and CR demonstrated similar moderate diagnostic capability in detecting mild HOA compared to MRI, and both the US sum score and KL grades were associated with increased hip pain. With US and CR demonstrating different aspects of HOA, our findings support the complementary role of US in evaluating patients with suspected HOA.

## Graphical abstract

A moderate‐sized femoral neck osteophyte (white arrow) in US (A), anteroposterior CR (B), and MRI (C). Additional osteoarthritis‐related changes (arrowheads) are visible in CR and MR, but not in US. US and CR demonstrated similar moderate diagnostic capability to detect mild hip osteoarthritis and were associated with increased hip pain.

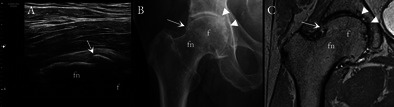

## 1 Introduction

Osteoarthritis (OA) is a common musculoskeletal disease characterized by synovial joint damage and foci of articular cartilage loss, subchondral bone change, narrowing of the articular space, osteophyte formation, and associated synovitis. OA is a leading cause of adult chronic pain and long‐term disability, causing significant personal and societal burden (GBD 2021 Osteoarthritis Collaborators 2023).

The hip joint is the third most common site for OA. The global incidence of symptomatic and conventional radiography (CR)‐confirmed hip OA (HOA) has been estimated to reach approximately 62.6 million in 2050—an increase of 78.6% from 2020 (GBD 2021 Osteoarthritis Collaborators 2023). Established risk factors of HOA are high body mass index (BMI) (GBD 2021 Osteoarthritis Collaborators 2023), cam deformity, and hip dysplasia (Saberi Hosnijeh et al. 2018; Saberi Hosnijeh et al. 2017; Gala et al. 2016; Agricola et al. 2013) as well as genetics (Boer et al. 2021). HOA has also been associated with manual labor (Harris and Coggon 2015) and high‐impact sports (Agricola et al. 2013; Vigdorchik et al. 2017) and its prevalence increases sharply with aging (GBD 2021 Osteoarthritis Collaborators 2023). With the global obesity epidemic (NCD Risk Factor Collaboration (NCD‐RisC) 2017) and aging populations, healthcare costs related to the treatment of OA have increased significantly. The most significant direct costs are related to knee and hip joint replacement procedures, which are being done to an ever wider patient population (Hunter et al. 2014; Hunter and Bierma‐Zeinstra 2019).

In terms of imaging, CRs are most commonly used in evaluating HOA patients, enabling the direct assessment of bony features of OA such as osteophytes and subchondral cysts and sclerosis, and the indirect assessment of articular cartilage status by visualizing the joint space width. CRs are unable to directly illustrate cartilage and soft tissue damage, and several features of HOA such as labral pathology and bone marrow lesions are not visible in them. Patient positioning and superimposition also pose a technical and an interpretation‐affecting challenge (Gold et al. 2015). The advantage of magnetic resonance imaging (MRI) is the superior visualization of soft tissue structures, but its price and availability somewhat limit its use (Sudula 2016). The applicability of ultrasound (US) in HOA diagnostics has increased as a result of the development of US devices. The use of US is supported by its availability and relative affordability, as well as the ability to evaluate soft tissue structures that CR does not recognize. An additional benefit is that US is an ionizing radiation‐free study and can identify more superficial bony changes of the hip joint (Sudula 2016; Qvistgaard et al. 2006).

The challenge of the usability of US has been considered to be the lack of repeatability due to operator dependent factors. The shape and the location of the hip joint, in turn, pose challenges to the ability of the US to reach the deepest parts of the joint. Preliminary results have been obtained on the reproducibility and usefulness of US research, but more research is still warranted (Qvistgaard et al. 2006; Oo et al. 2018; Nevalainen et al. 2023; Nevalainen et al. 2020; Clausen et al. 2020). Accordingly, comparative studies between US and other imaging modalities are still limited for the time being (Qvistgaard et al. 2006; Oo et al. 2018; Nevalainen et al. 2023).

The purpose of this study was to evaluate the diagnostic performance of the US against CR in mild HOA using MRI as the reference standard. Moreover, pain association against the developed US sum score and CR‐determined KL grades were studied.

## 2 Materials and Methods

### 2.1 Patients

In this prospective study, 60 patients with at least unilateral hip pain and clinically suspected HOA were initially recruited from the hospital's patient records based on hip CR (imaged between January and June 2022) where at least unilateral Kellgren‐Lawrence (KL) (Kellgren and Lawrence 1957) grade 2 HOA was identified. Subsequently, all patients underwent imaging with both hips using US and MRI, and patients completed a visual analogue scale (VAS) pain questionnaire on each hip. For each patient, US and MRI examinations and pain questionnaires were done on a single examination visit between March and September 2022. During the examination visit subjects self‐reported their body height and weight, and body mass index (BMI) was calculated. The complete inclusion criteria for the study were: (1) imaged hip CR between January and June 2022 with hip pain and suspected HOA in the referral, (2) KL grade 2 HOA in CRs in one or both hip joints, (3) willingness to participate in the study and (4) completed bilateral hip CR, US and MRI examinations. The exclusion criteria for the study were: (1) evidence of fracture or malignancy in clinical imaging, (2) diagnosed musculoskeletal disorders severely affecting either hip joint in clinical imaging (e.g., rheumatoid arthritis), (3) previous hip surgery and (4) any contraindications to MRI. The initial evaluation of CRs was done by a fellowship‐trained musculoskeletal radiologist with 10 years of experience (MTN). In the patient recruitment workflow, a total of two patients were excluded: one patient due to a recent fracture and one patient due to refusal of MRI.

This study followed the Declaration of Helsinki ethical principles and was performed under the ethical permission (42/2022) issued by the Northern Ostrobothnia Hospital District Ethics Committee. Written informed consent was obtained from all participants.

### 2.2 US Imaging

A single experienced sonographer with over 10 years of experience performed all the US studies. Samsung RS85 US device with linear 2–14 MHz transducer was used. Patients were imaged in supine position with the ipsilateral hip and knee joints in extension. US was used to assess the presence of osteophytes, femoral cartilage damage, the shape of the femoral head and the amount of hip effusion. Osteophytes were assessed from the anterolateral femur and the superolateral acetabulum separately; due to low US‐visibility, inferomedial femoral and acetabular osteophytes were not assessed by US in this study. Osteophytes were classified as follows: Grade 0 = no osteophyte, Grade 1 = minimal osteophyte, Grade 2 = definite osteophyte. Cartilage was assessed at the head of the femur, dividing the findings into three categories: Grade 0 = normal, Grade 1 = a local focus of possibly thinned cartilage, Grade 2 = a local focus of clear cartilage damage. The femur shape was divided into three categories as follows: Grade 0 = normal, Grade 1 = slightly abnormal, Grade 2 = flat/collapsed. The amount of effusion was measured from the anterior joint recess as the largest distance between the anterior femoral neck and the joint capsule. The US examination was done before MRI, and subsequently the sonographer was blinded to MRI but not to CRs.

### 2.3 CR Imaging

CR of the hips was used to assess osteophytes, joint space narrowing, femoral head shape, and KL grades on pelvis AP projection (Kellgren and Lawrence 1957). Superolateral and inferomedial osteophytes were assessed from the femoral neck and acetabulum with the following classification: Grade 0 = no osteophytes, Grade 1 = small/minimal osteophyte, Grade 2 = a definite osteophyte. The narrowing of the joint space was assessed superolaterally and inferomedially and classified as: Grade 0 = normal joint space, Grade 1 = slight/minor narrowing, Grade 2 = obvious narrowing. The shape of the femur head was assessed as follows: Grade 0 = normal, Grade 1 = slightly abnormal, Grade 2 = clearly deformed. A fellowship‐trained musculoskeletal radiologist with 10 years of experience (MTN) performed the evaluation and was blinded to MRI and US.

### 2.4 MR Imaging

Hip MRI was performed with a 3T MRI scanner (“Skyra,” Siemens Healthineers, Erlangen, Germany) with a body coil. Similar MRI protocols were used for all subjects with the following MRI sequences: Short Tau Inversion Recovery sequence with coronal reconstruction and isotropic (i.e., 3D) turbo spin echo proton density weighted sequence with coronal reconstruction (Table 1).

**TABLE 1:** Magnetic resonance imaging sequence parameters: cor; coronal, FOV; field‐of‐view, PD; proton density, SPACE; Sampling Perfection with Application optimized Contrast using different flip angle Evolution, STIR; Short Tau Inversion Recovery, TE; echo time, TR; repetition time.

	TR	TE	Slice thickness	Spacing	Flip angle	Matrix size
STIR cor	5080 ms	30 ms	3.0 mm	3.9 mm	140°	512 × 280
PD cor SPACE	1100 ms	23 ms	0.80 mm	0.8 mm	120°	448 × 294

Hip MRI images were assessed using the Hip Osteoarthritis MRI Scoring System (HOAMS), a semiquantitative MRI scoring system with adequate reliability (Roemer et al. 2011). In HOAMS, several image features are scored with respect to their location and severity. For this study, osteophytes, cartilage lesions, signs of attrition (shape of the femoral head), and joint effusion were evaluated. Osteophytes of superolateral and anteromedial femoral neck and acetabulum were graded as follows: Grade 0 = no osteophytes, Grade 1 = equivocal or questionable osteophyte, Grade 2 = small beak‐like definite osteophyte, Grade 3 = intermediate‐size osteophyte, Grade 4 = proliferative large osteophyte. Cartilage regions were evaluated in the nine HOAMS‐defined subregions as follows: Grade 1 = focal partial‐thickness defect 25% of the subregional area, Grade 2 = focal full thickness defect 25% of the subregional area, Grade 3 = several partial thickness defects or single but larger superficial defect > 25% of the subregional area, Grade 4 = several large full thickness defects or single full thickness defect > 25% of the subregional area. Attrition was defined as definite flattening or asphericity of the femoral head or the acetabular curvature in the weight‐bearing part of the joint and graded as either absent or present. Joint effusion was graded as follows: Grade 0 = no effusion, Grade 1 = possible or small effusion at most, Grade 2 = definite effusion. Scoring was done by a board‐certified radiologist with 5 years of experience (AK) after training and calibration sessions with a board‐certified fellowship‐trained musculoskeletal radiologist with 10 years of experience (MTN).

## 3 Statistical Analysis

Patient and imaging data are presented in descriptive statistics as counts, percentages, means, medians, and ranges. US, CR, and MRI grades were considered categorical ordinal data except US‐measured hip effusion was regarded as a continuous variable. A Spearman correlation coefficient was calculated to assess the correlation between the US sum score and KL grade. Linear regression analysis was applied to study the association between imaging findings and pain.

The diagnostic performance of US and CRs compared to MRI was assessed with contingency tables using dichotomized data. Osteophyte grades 0–1 were regarded as negative findings for all imaging modalities, and grade 2 in US and CR and grades 2–4 in MRI were regarded as positive findings, respectively. US‐identified cartilage lesion and CR‐identified joint space narrowing grades 0–1 were regarded as negative findings, and grade 2 was regarded as a positive finding. A cutoff of 7 mm was used for joint effusion (Koski et al. 1989). US‐identified anterolateral femoral neck and superolateral acetabular osteophytes, femoral head deformity, and joint effusion were compared to their respective parameters in MRI. CR‐identified anterolateral and inferomedial femoral neck and acetabular osteophytes and femoral head deformity were also compared to their respective parameters in MRI. For comparing US‐identified cartilage lesions and CR‐depicted joint space narrowing to MRI, several HOAMS‐defined MRI subregions were pooled to best depict the anterosuperior and inferomedial cartilage regions. Of the respective subregions, the single highest cartilage lesion grade was selected for dichotomization and analysis. Centra‐lateral, central‐central, and antero‐superior HOAMS MRI subregions were pooled to represent the anterosuperior cartilage region, and central‐medial, central‐inferior, and antero‐inferior HOAMS MRI subregions were pooled to represent the inferomedial cartilage region.

US sum score was formed from the dichotomized hip effusion, femoral cartilage damage, the presence of osteophytes at the femoral neck and acetabulum, and the shape of the femoral head. Each positive finding counted as a score of one, with the maximum sum score being five.

Statistical analyses were done using RStudio, version 4.3.3 (Posit PBC, Boston, Massachusetts, US).

## 4 Results

Complete bilateral imaging was available for 58 patients (females *n* = 41, 70.7%), totaling 116 hip joints. Mean delay between CRs and MRI and US examination was 106.2 days (median 89 and range 65–218 days). Mean patient age at the time of MRI and US examinations was 66 years (median 68.1 and range 42.8–82.2 years). Hip KL classification was grade 0 in 1 (0.8%), grade 1 in 28 (24.1%), grade 2 in 66 (56.9%), grade 3 in 20 (17.2%), and grade 4 in 1 (0.8%) hip joints, respectively. Complete height and weight information was available for 53 (91.4%) patients. Mean BMI was 27.6 kg/m^2^, classified as overweight (median 27.2 kg/m^2^ and range 13.0–42.2 kg/m^2^). According to WHO‐defined BMI categories (WHO, n.d.), 2 (3.8%) of the patients were underweight (< 18.5 kg/m^2^), 16 (30.2%) were normal weight (≥ 18.5 kg/m^2^), 19 (35.8%) were overweight (≥ 25 kg/m^2^), and 16 (30.2%) were obese (≥ 30 kg/m^2^).

**FIGURE 1:**
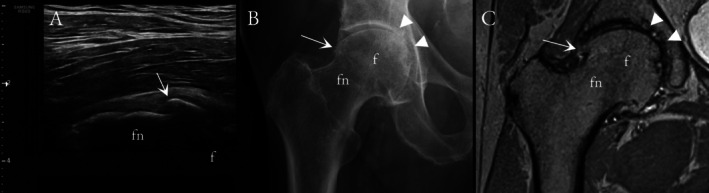
A 69‐year‐old male with a moderate‐sized femoral neck osteophyte (white arrow) visualized in longitudinal US (A), anteroposterior CR (B), and coronal PD‐weighted MRI plane (C). The osteophyte is well delineated in both US and CR. Additional medial joint space narrowing and subchondral bone changes (arrowheads) are visible in CR and MR, but not reachable by US. f, femoral head; fn, femoral neck.

**FIGURE 2:**
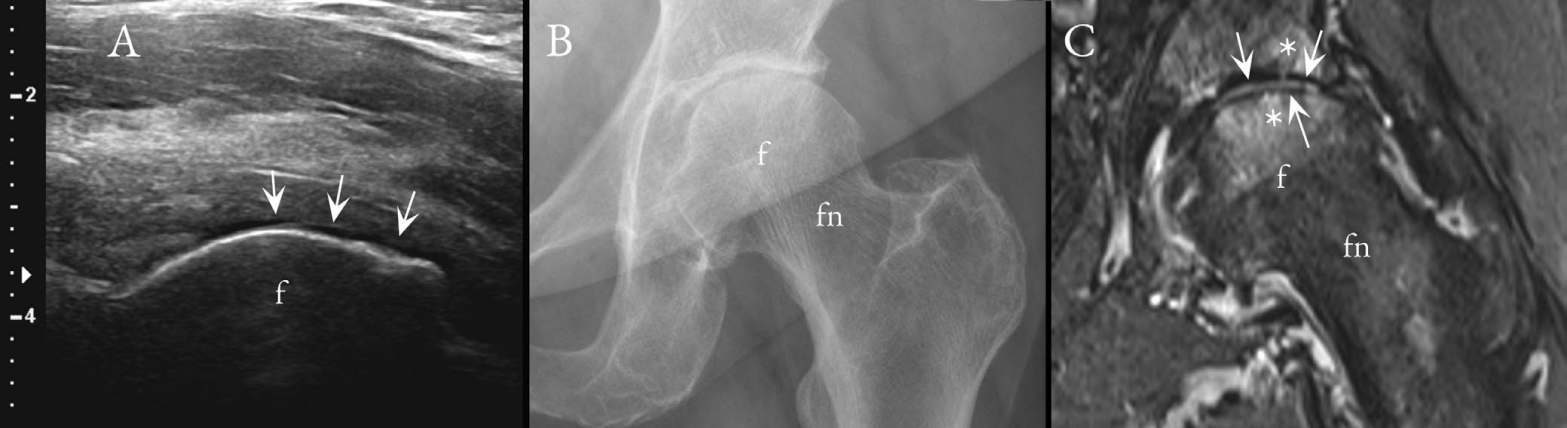
A 65‐year‐old woman with local wearing of the superolateral anterosuperior articular cartilage of the femoral head. In longitudinal US (A), cartilage wear is visualized as heterogenic intrachondral signal, cartilage thinning, varying cartilage thickness, and visualized local areas of cartilage defect. In this case, cartilage was evaluated as grade 2 (clear cartilage damage) in US. Anteroposterior CR (B) shows superolateral joint space narrowing. Coronal STIR MRI sequence (C) shows a large superior and superolateral cartilage defects and associated subchondral bone marrow lesion, indicative of full‐thickness cartilage damage. f, femoral head; fn, femoral neck.

**FIGURE 3:**
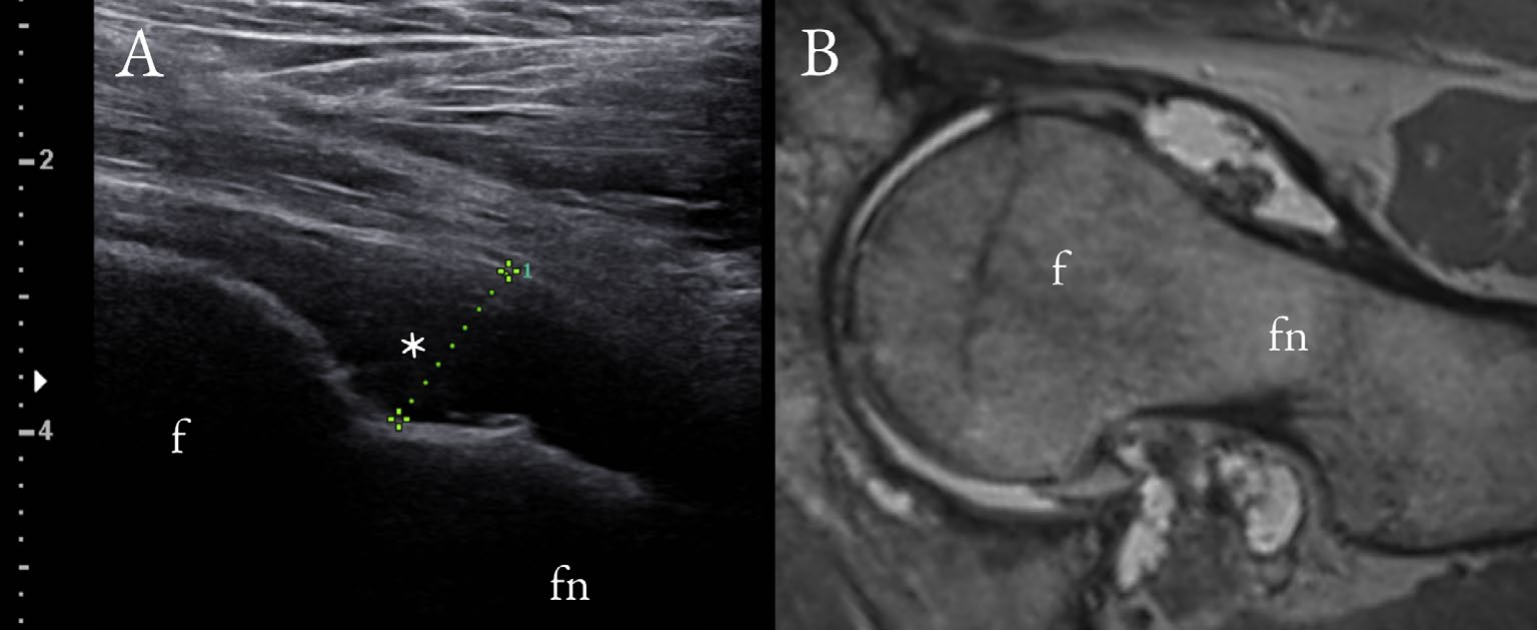
A 68‐year‐old man with moderate‐large hip joint effusion visualized in longitudinal US (asterisk) (A). PD‐weighed MRI sequence in oblique axial reformation (B) also shows clear hip effusion (black asterisk) as well as synovial hypertrophy, thought to be secondary to osteoarthritis. No evidence of hip effusion was seen in anteroposterior CR (not shown).

**FIGURE 4:**
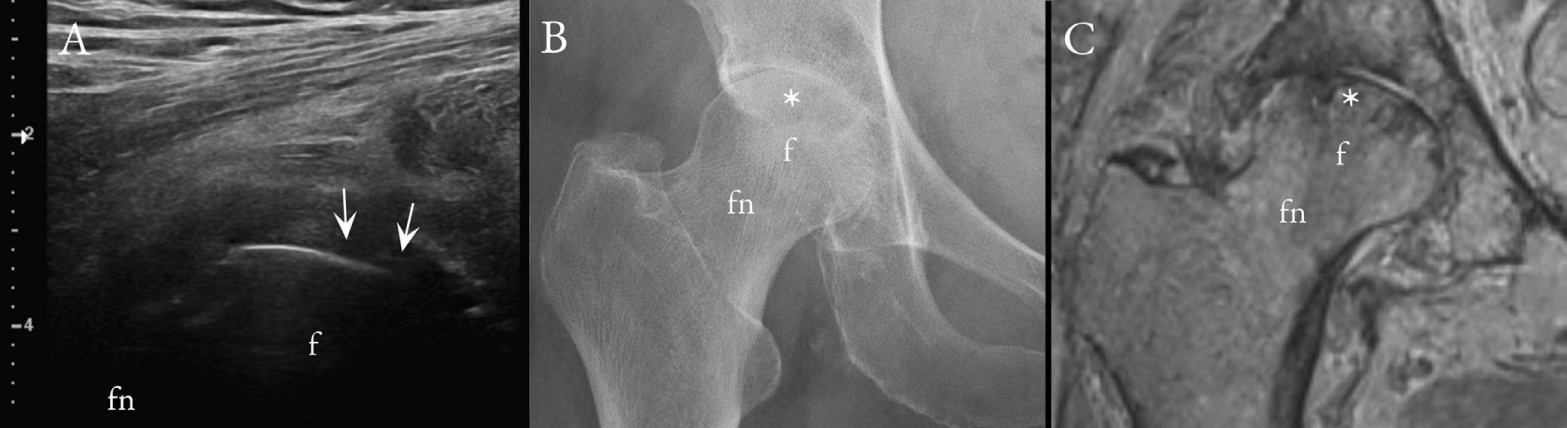
A 75‐year‐old woman with loss of normal femoral head sphericity and visibly flattened subchondral bone surface in longitudinal US (A). Anteroposterior CR (B) shows local subchondral lucency in the superior joint space (asterisk) and slight femoral head shape abnormality. Coronal PD‐weighted MRI (C) shows attrition‐associated subchondral cysts, sclerosis, and flattening of the femoral head. f, femoral head; fn, femoral neck.

Overall, US and CR showed only moderate diagnostic performance to detect mild HOA. In US, the sensitivities to detect osteophytes (Figure 1) varied between 62% and 74%, specificities between 53% and 86%, and accuracies between 61% and 82%; for the CR, the respective values were 56%–92%, 69%–93%, and 75%–87%. For cartilage lesions (Figure 2), the US showed 30% sensitivity, 89% specificity, and 65% accuracy, whereas CR depicted 30%–88% sensitivity, 32%–95% specificity, and 41%–68% accuracy. Example images of US‐identified hip effusion and femoral head deformity are shown in Figures 3 and 4. Tables 2 and 3 sum up the diagnostic performance of US and CR to detect hip OA when using MRI as the reference standard. In Tables 4 and 5, it can be seen that there is virtually no difference between US and CR to detect HOA; only when looking at the osteophytes of the anterosuperior acetabulum, the US seems to confabulate the presence of osteophytes as compared to CR. Correlation between the developed US sum score and KL grades was only moderate (*r* = 0.449). Last, in the regression analysis to evaluate pain association, the US sum score showed an OR = 1.725 (CI 1.169–2.546), and KL grades an OR = 2.058 (CI 1.038–4.082).

**TABLE 2:** Counts of hip joint US and MRI findings and the diagnostic performance with 95% Confidence Intervals of US with MRI as the reference standard.

	TP/N1	Sens.	TN/N2	Spec.	N3	Acc.	PPV	NPV
Osteophytes								
Anterosuperior femoral neck	10/16	62 (35–85)	81/94	86 (78–92)	91	82 (74–89)	43 (23–66)	93 (86–97)
Anterosuperior acetabulum	31/42	74 (58–86)	36/68	53 (40–65)	67	61 (51–70)	53 (40–65)	53 (40–65)
Cartilage								
Anterosuperior femoral head	14/46	30 (18–46)	57/64	89 (79–95)	71	65 (55–73)	67 (43–85)	64 (53–74)
Femoral head deformity	1/3	33 (1–91)	104/107	97 (92–99)	105	95 (90–99)	25 (1–81)	98 (93–100)
Effusion	8/13	62 (32–86)	72/97	74 (64–83)	80	73 (63–81)	24 (11–42)	94 (85–98)

Abbreviations: Acc., accuracy; N1, number of MRI positive findings; N2, number of negative MRI findings; N3, number of cases where MRI agrees with US; NPV, negative predictive value; PPV, positive predictive value; Sens., sensitivity; Spec., specificity; TN, number of negative US findings; TP, number of US positive findings.

**TABLE 3:** Counts of hip joint CR and MRI findings and the diagnostic performance with 95% Confidence Intervals of CR with MRI as the reference standard.

	TP/N1	Sens.	TN/N2	Spec.	N3	Acc.	PPV	NPV
Osteophytes								
Anterosuperior femoral neck	10/16	62 (35–85)	77/94	82 (73–89)	87	79 (70–86)	37 (19–58)	93 (85–97)
Anterosuperior acetabulum	35/42	83 (69–93)	47/68	69 (57–80)	82	75 (65–82)	62 (49–75)	87 (75–95)
Inferomedial femoral neck	10/18	56 (31–78)	86/92	93 (86–98)	96	87 (80–93)	62 (35–85)	91 (84–96)
Inferomedial acetabulum	12/13	92 (64–100)	82/97	85 (76–91)	94	85 (77–91)	44 (25–65)	99 (93–100)
Cartilage								
Anterosuperior femoral head	14/46	30 (18–46)	61/64	95 (87–99)	75	68 (59–77)	82 (57–96)	66 (55–75)
Inferomedial femoral head	15/17	88 (64–99)	30/93	32 (23–43)	45	41 (32–51)	19 (11–30)	94 (79–99)
Femoral head deformity	1/3	62 (35–85)	106/107	86 (78–92)	107	97 (92–99)	43 (23–66)	93 (86–97)

Abbreviations: Acc., accuracy; NPV, negative Predictive value; N1, number of MRI positive findings; N2, number of negative MRI findings; N3, number of cases where MRI agrees with CR; PPV, positive predictive value; Sens., sensitivity; Spec., specificity; TP, number of CR positive findings; TN, number of negative CR findings.

**TABLE 4:** The diagnostic performance of US and CR compared to positive MRI findings.

US finding vs. radiography finding	MRI+	US+/CR+	US+/CR−	US−/CR+	US−/CR−	McNemar	*p*
Osteophytes							
Anterosuperior femoral neck	16	8 (50.0)	2 (12.5)	2 (12.5)	4 (25.0)	0	1
Anterosuperior acetabulum	42	25 (59.5)	6 (14.3)	10 (23.8)	11 (26.2)	0.56	0.4533
Cartilage							
Anterosuperior femoral head	46	8 (17.4)	6 (13.0)	6 (13.0)	26 (56.5)	0	1
Femoral head deformity	3	1 (33.3)	0 (0.0)	0 (0.0)	2 (66.7)	0	NA

*Note:* Data are presented as count (*n*) and percentage (%).

Abbreviations: MRI+, total number of MRI positive findings; US+/CR+, number of MRI positive, US positive, CR positive findings; US+/CR−, number of MRI positive, US positive, CR negative findings; US−/CR+, number of MRI positive, US negative, CR positive findings; US−/CR−, number of MRI positive, US negative, CR negative findings.

**TABLE 5:** The diagnostic performance of US and CR compared to negative MRI findings.

US finding vs. radiography finding	MRI−	US+/CR+ *N* (%)	US+/CR− *N* (%)	US−/CR+ *N* (%)	US−/CR− *N* (%)	McNemar	*p*
Osteophytes							
Anterosuperior femoral neck	94	4 (4.3)	9 (9.6)	13 (13.8)	68 (72.3)	0.41	0.52
Anterosuperior acetabulum	68	14 (20.6)	18 (26.6)	7 (10.3)	29 (42.6)	4	0.045
Cartilage							
Anterosuperior femoral head	64	0 (0.0)	7 (10.9)	3 (4.7)	54 (84.4)	0.90	0.3428
Femoral head deformity	107	1 (0.9)	2 (1.9)	0 (0.0)	104 (97.2)	0.50	0.48

*Note:* Data are presented as count (*n*) and percentage (%).

Abbreviations: MRI−, total number of MRI negative findings; US+/CR+, number of MRI negative, US positive, CR positive findings; US+/CR−, number of MRI negative, US positive, CR negative findings; US−/CR+, number of MRI negative, US negative, CR positive findings; US−/CR−, number of MRI negative, US negative, CR negative findings.

## 5 Discussion

In this study, the diagnostic performance of US and CRs in detecting structural findings of HOA was compared to MRI in referred patients with clinical suspicion of HOA. Our results demonstrate that both the US and CR depict only moderate ability to detect mild HOA changes. Interestingly, although the developed US sum score and KL grades exhibited rather poor overall correlation, they were both independently associated with increased hip pain.

The deep anatomical location of the hip joint seems to be a central hindrance to more widespread adoption of US as a primary diagnostic tool for HOA, and only a few studies have been published on the matter (Nevalainen et al. 2023). Whereas hip US excels in image‐guided interventions (Hoeber et al. 2016) and the visualization of extra‐articular structures (Rosenberg et al. 2024; Iagnocco et al. 2012), the evaluation of intra‐articular pathology and acetabular osteophytes remains limited by variable reproducibility and inconsistent correlation with clinical symptoms (Nevalainen et al. 2023). The sonographic visibility of the hip joint is most certainly affected by patient size and subsequently BMI, which is surprisingly rarely reported in the current literature. US shows promise in evaluating certain aspects of hip joint pathology, particularly osteophytes and femoral head convexity (Qvistgaard et al. 2006; Nevalainen et al. 2020; Clausen et al. 2020). In late‐stage HOA patients scheduled for total hip arthroplasty, US performed similarly to CRs in detecting osteophytes and was superior to CRs in detecting femoral head deformity with moderate to excellent inter‐rater reliability. The surgical sample was used as the reference standard (Nevalainen et al. 2020). Whereas US evaluation of hip effusion also shows good to excellent inter‐rater reliability (Qvistgaard et al. 2006; Clausen et al. 2020), and US‐identified large joint effusion is reportedly correlated with rapidly destructive osteoarthritis (Birn et al. 2014), the effectiveness of US in ruling out hip effusion in the adult hip seems to also be limited (Weybright et al. 2003). The femoral articular cartilage surface has been evaluated with US before with some difficulty (23.9% of excluded hip joints due to irregular cartilage surface deemed imprecise to evaluate) and moderate to substantial agreement (Clausen et al. 2020).

In our study, patients were imaged with US and CRs as well as with MRI for the reference standard. The US examination focused on evaluating the less investigated mostly intra‐articular structures associated with HOA: femoral neck and acetabular osteophytes, visible articular cartilage lesions of the femoral head, femoral head deformity as well as hip joint effusion. By current knowledge, all these structural features are independently associated with hip pain on MRI, although the number of publications on the matter is limited (Fang et al. 2024). In our results, US and CRs showed similar moderate diagnostic performance in detecting osteophytes and cartilage loss (with CRs evaluating the loss of joint space width). The developed US sum score and CR‐evaluated KL grades showed poor overall correlation. This finding is similar to Qvistgaard et al. where the developed US score was reproducible and predictive of VAS scores but weakly correlated to patient KL grades (Qvistgaard et al. 2006), suggesting that US and CRs identify different characteristics of HOA. Hip effusion is typically unevaluable by CR, whereas its detection with US is straightforward. CR enables the evaluation of osteoarthritis‐associated subchondral bone changes, whereas US can potentially increase the detection rate of small acetabular or femoral neck osteophytes. As only a few MRI‐confirmed femoral head deformities and some hip joint effusions were present in our study population with high specificity and negative predictive value in US evaluation, a significant number of false positives were not produced in these clinically concerning findings. As such, US could be used to complement CRs in evaluating also the intra‐articular hip joint structures, for example, adjunct to a primary or secondary health care visit. Added US examination would also enable the evaluation of extra‐articular hip structures such as tendon and muscle injuries as well as bursae (Martinoli et al. 2012).

Similar complementary roles between US and CR in evaluating structural osteoarthritis features have been previously reported in other joints. Most research and examples can be found in the knee joint (Nevalainen et al. 2023). The medial meniscus can be directly visualized with US in both supine and weight‐bearing conditions (Uusimaa et al. 2024; Karpinski et al. 2019), whereas CRs are limited to evaluating joint space narrowing, which is affected by both cartilage damage and meniscus status. Local cartilage damage of the knee can also be diagnosed with US (Roemer et al. 2022) on visible articular cartilage surfaces (Kauppinen et al. 2021), US is very sensitive in the detection of knee joint effusion and is also able to evaluate for features of synovitis. CR, on the other hand, provides a great general estimation of bony anatomy, osteophytes, and osteoarthritis‐associated subchondral bone changes, and is the most widely utilized imaging modality for knee osteoarthritis (Kauppinen et al. 2021). Although CR is typically used for evaluating osteophytes, US has been found to be more sensitive in the detection of small tibiofemoral osteophytes, potentially subclinical in their nature (Nevalainen et al. 2023; Roemer et al. 2022). Modern US techniques could further improve the utility of US in the routine evaluation of knee osteoarthritis patients in the future, such as shear wave elastography for cartilage (Yokuş et al. 2021) and superb microvascular imaging for synovitis (Oo et al. 2020). The complementary role of US is also well established in the hand joints, where multiparametric US is routinely used, especially in the evaluation of synovitis and bone erosions—features that are undetectable in CR in the early phase (Nevalainen et al. 2023).

There are some limitations to our study. First, our study population exceeded the WHO definition of normal weight, and a significant portion of patients were categorized as obese. As US visibility of the hip joint is majorly affected by the patient weight, this could in part explain the modest performance of US in our study population. The diagnostic performance of US in different BMI categories was not evaluated, as the number of patients was overall relatively low, but this would also be an exciting avenue for future research. Study population BMIs seem to not be systematically reported in the current literature on hip US examinations. Second, as only pelvis AP CR was applied for the radiographic assessment of HOA, the lack of lateral projection might explain the poor performance of CR, too. Additionally, on MRI we used an isotropic proton density weighted sequence with very small slice thickness, enabling multiplanar reconstruction and meticulous detection of small osteophytes. Third, as HOAMS has been designed for standard non‐arthrography MRI, acetabular and femoral articular cartilage surfaces are scored together as they are indistinguishable from one another without intra‐articular contrast agent. According to current understanding, US is only capable of identifying femoral articular cartilage lesions, and cartilage lesions on the acetabular surface would go unnoticed. For future research, MR‐arthrograms could be a more representative reference standard. Fourth, no intra‐reader or inter‐reader data is presented; however, previous publications have shown hip US to be a reproducible imaging examination with generally good to excellent intra‐rater and acceptable inter‐rater reliability (Qvistgaard et al. 2006; Oo et al. 2018; Nevalainen et al. 2023; Nevalainen et al. 2020; Clausen et al. 2020). To mitigate the risk of error in our single operator approach, only clear US‐identified cartilage damage (grade 2) was regarded as positive findings, whereas potentially more ambiguous possible cartilage lesions (grade 1) were considered as negative findings. As US is in general considered a highly operator‐dependent modality, especially in small joints (Oo et al. 2018), future research could benefit from multiple US evaluations or, alternatively, the use of cine loops for single or multiple retrospective evaluations.

## 6 Conclusions

US and CR demonstrated moderate diagnostic capabilities to detect mild hip OA, and there were no differences within modalities' performances compared to MRI. The developed US sum score showed a rather weak correlation to KL grades; however, both the US sum score and KL grades were independently associated with increased hip pain. This would seem to indicate that US and CR demonstrate different aspects of HOA. Our study adds to the limited comparative literature on hip US and supports its role as a complementary tool to CR in the assessment of hip osteoarthritis.

## Conflicts of Interest

The authors declare no conflicts of interest.

## Data Availability

The data that support the findings of this study are available from the corresponding author upon reasonable request.
